# Rapid SERS Assay for Determination of the Opioid Fentanyl Using Silver-Coated Sharply-Branched Gold Nanostars

**DOI:** 10.1007/s00604-023-06172-5

**Published:** 2024-01-22

**Authors:** Supriya Atta, Aidan J. Canning, Tuan Vo-Dinh

**Affiliations:** a Fitzpatrick Institute for Photonics, Duke University, Durham, NC 27708, USA.; b Department of Biomedical Engineering, Duke University, Durham, NC 27708, USA.; c Department of Chemistry, Duke University, Durham, NC 27708, USA.

**Keywords:** Gold nanostars, silver coated gold nanostars, bimetallic, fentanyl, SERS, sensing

## Abstract

Herein, we demonstrated a high-throughput surface-enhanced Raman scattering (SERS)-sensing platform for FNT detection in human urine without any sample preparation. The sensing platform was based on plasmonics-active silver-coated sharply branched gold nanostars (SGNS). The effect of silver thickness was investigated experimentally and theoretically, and the results indicated that SERS enhancement was maximum at an optimum silver thickness of 45 nm on the sharply spiked SGNS. The proposed high-throughput SERS platform exhibited ultrahigh SERS sensitivity and excellent enhancement uniformity for a model analyte, i.e., crystal violet. Moreover, the SERS-sensing platform demonstrated good detection sensitivity of FNT spiked in human urine samples with two differential linear responses ranging from 2 to 0.2 μg/mL and 0.1 μg/mL to 100 pg/mL, with a detection limit as low as 10.02 pg/mL. The spiked human urine samples show satisfactory recovery values from 92.5 % to 102 % with relative standard deviations (RSD) of less than 10 %. In summary, the high-throughput performance of the proposed microplate-based SERS platform demonstrated great potential for rapid low-cost SERS-based sensing applications.

## Introduction

Fentanyl (FNT), one of the most effective synthetic opioid medications, is widely prescribed and used for pain medication and surgical analgesia in patients with chronic pain and cancer[1–3]. Although FNT is a powerful pain reliever, it can lead to abuse and addiction because of its strong euphoria, relaxation, and unconscious effects. The Centers for Disease Control and Prevention reported 71,238 deaths in 2021 from FNT overdoses in the United States[4]. Therefore, it is important to identify accurately and rapidly to prevent the misuse of this psychoactive substance. Traditionally, gas chromatography-mass spectrometry (GC-MS)[5], high-performance liquid chromatography (HPLC)[6], and enzyme-linked immunosorbent assay (ELISA)[7] have been used for the trace detection of FNT. Although these methods have high accuracy, they are limited to research laboratories, which require expensive reagents, time-consuming pretreatment steps, and highly skilled expertise in chromatography, thereby making the analysis unsuitable for time-efficient routine in-field test applications.

Recently, surface-enhanced Raman scattering (SERS) spectroscopy has been attracting attention as an alternative analytical technique for sensitive and label-free detection of a wide range of molecular analytes, including illegal drugs, e.g., FNT [8–11]. Almost four decades ago, our laboratory first introduced SERS as an analytical tool and has developed this technique for various sensing applications[12, 13]. Several strategies were reported for the development of processes regarding the rapid and sensitive SERS detection of FNT, which can be classified into two general categories: (i) metal nanostructure-supported solid substrates, e.g., capillary-based substrate [14], adhesive flexible plasmonic patches [15], paper-based substrates[16], and (ii) solution-based SERS substrate[17]. Although solid substrates have several advantages over solution-based substrates, such as better sensitivity and specificity, they are time-consuming with regard to preparation and suffer from inhomogeneous nanoparticle distribution, which limits the point-of-need practical application[18, 19]. Consequently, the solution-based method can help make the SERS platform a cost-effective high-throughput device enabling the rapid identification of analytes with microfluidic devices and microplate readers[20]. However, the clinical use of solution-based SERS platforms has limitations, such as inconsistent molecular absorption, competition between endogenous metabolites and target analytes, and random aggregation of nanoparticles owing to surface fouling caused by proteins present in biological fluids[21].

To achieve sensitive SERS signals in a solution-based SERS assay, one of the effective solutions is to design a gold nanoparticle system to generate ultrastrong electromagnetic fields. Over the past decades, considerable effort has been made to modify and manipulate the gold nanoparticle’s geometry to tailor electromagnetic properties[12]. Recently, the focus has been on anisotropic gold nanostars, which exhibit more potential for SERS applications than gold nanospheres, nanoshells, and nanorods because of the generation of highly localized electromagnetic fields named “hot spots” in the presence of sharp protruding spikes[22]. To achieve ever-brighter SERS, bimetallic gold-silver nanoparticles have been considered to use the high plasmonic effect of Ag and strong enhanced electromagnetic fields induced by the sharply branched gold nanostars[23]. Despite the well-known SERS enhancement properties of gold nanostars and bimetallic gold-silver nanoparticles, there is a scarcity of high-yield strategies and clear design principles of the nanoplatforms to achieve maximum SERS enhancement. Therefore, silver-coated SGNS morphology needs to be investigated via experimentation and simulation to accurately predict the desired morphology to achieve maximum electric field enhancement.

Herein, we presented a high-throughput microplate reader-based SERS platform for FNT detection using silver-coated SGNSs. To understand the effect of silver thickness on sharply spiked gold nanostars for SERS enhancement, we employed finite element method (FEM) simulations, which indicated that the SGNS-45 configuration exhibited maximum SERS enhancement. We further investigated the reproducibility, stability, and capability to detect FNT spiked in an aqueous solution and human urine. The results indicated that the proposed microplate-based SERS platform exhibited remarkably high sensitivity and reproducibility compared with conventional reported SERS substrates, which allowed us to detect FNT in aqueous and human urine samples without any sample pretreatment. Furthermore, the percentage content of FNT adulterated in codeine and a mixture of opioids (FNT, codeine, and morphine) were successfully identified, indicating the multiplex capability of the proposed SERS method.

## Experimental section

### Materials and Characterization

Chloroauric acid (HAuCl_4_), L-ascorbic acid, silver nitrate (AgNO_3_, 99.8%) hydrochloric acid (HCl), trisodium citrate (Na_3_C_6_H_5_O_7_), fentanyl (1mg/mL), codeine (1mg/mL), and morphine (1mg/mL) were purchased from Sigma-Aldrich. Pooled human urine sample was purchased from Innovative Research. We have also used a healthy volunteer’s urine from Duke university. The STEM images were obtained by using Aberration Corrected STEM Thermo-Fisher Titan 80–300. UV-vis spectra were obtained by using a Shimadzu UV-3600i spectrometer with cuvettes of 1 cm path length.

### Synthesis of SGNS and Raman measurements

The synthesis method of SGNS and Raman measurements were discussed in detail in the Supplementary Information.

### Finite Element Modeling of SGNS Nanostructures

All nanoparticle simulations were conducted using the wave optics package within COMSOL Multiphysics 6.0. The simulation domain spanned 900 nm in all 3 dimensions, and the simulation environment was fit with the optical properties of water described by Diamon and Masumura [24]. For all simulated nanoparticle morphologies, GNS/SGNS gold core radius was held constant at 35 nm, and a branch length of 90 nm. The Johnson and Christy model was used to fit the optical properties of gold and silver.[25] A total of 6 different silver layer thicknesses over the gold nanoparticle core were examined: 0 nm, 10 nm, 20 nm, 30 nm, 45 nm, and 50 nm. The meshing of all simulated domains was physics-solver-controlled and extra fine. All SGNS models were exposed to a linearly polarized plane wave propagating in the x direction.

## Results and discussion

Bimetallic gold-silver nanostructures have been attracting attention as a promising SERS platform, which can integrate the advantages of Au and Ag within a nanostructure, where silver helps provide rich plasmonic modes and gold helps generate highly anisotropic nanostructures providing excellent field enhancement properties [26, 27]. Over the past few years, silver-coated gold nanostars have garnered considerable interest as an excellent SERS platform owing to the integration of the advantages of highly scattered silver coating and anisotropic sharp branches of gold to produce intense strong localized surface plasmon resonance (LSPR) named “hot spots”[28–30]. However, there have been limited studies on achieving the maximum SERS efficiency of a specific nanostar morphology, primarily because of the poor reproducibility of synthesis and unclear design principles. To investigate the potential of SGNSs as a SERS probe, we need to understand the mechanism to rationally link the plasmonic properties of the silver thickness on highly spiked GNSs to achieve the most effective SERS enhancement. In particular, we want to correlate the computationally predicted electromagnetic field enhancement of a specific morphology of silver-coated gold nanostars with experimentally observed SERS enhancement.

### Synthesis and characterization of SGNSs

**Figure 1.**
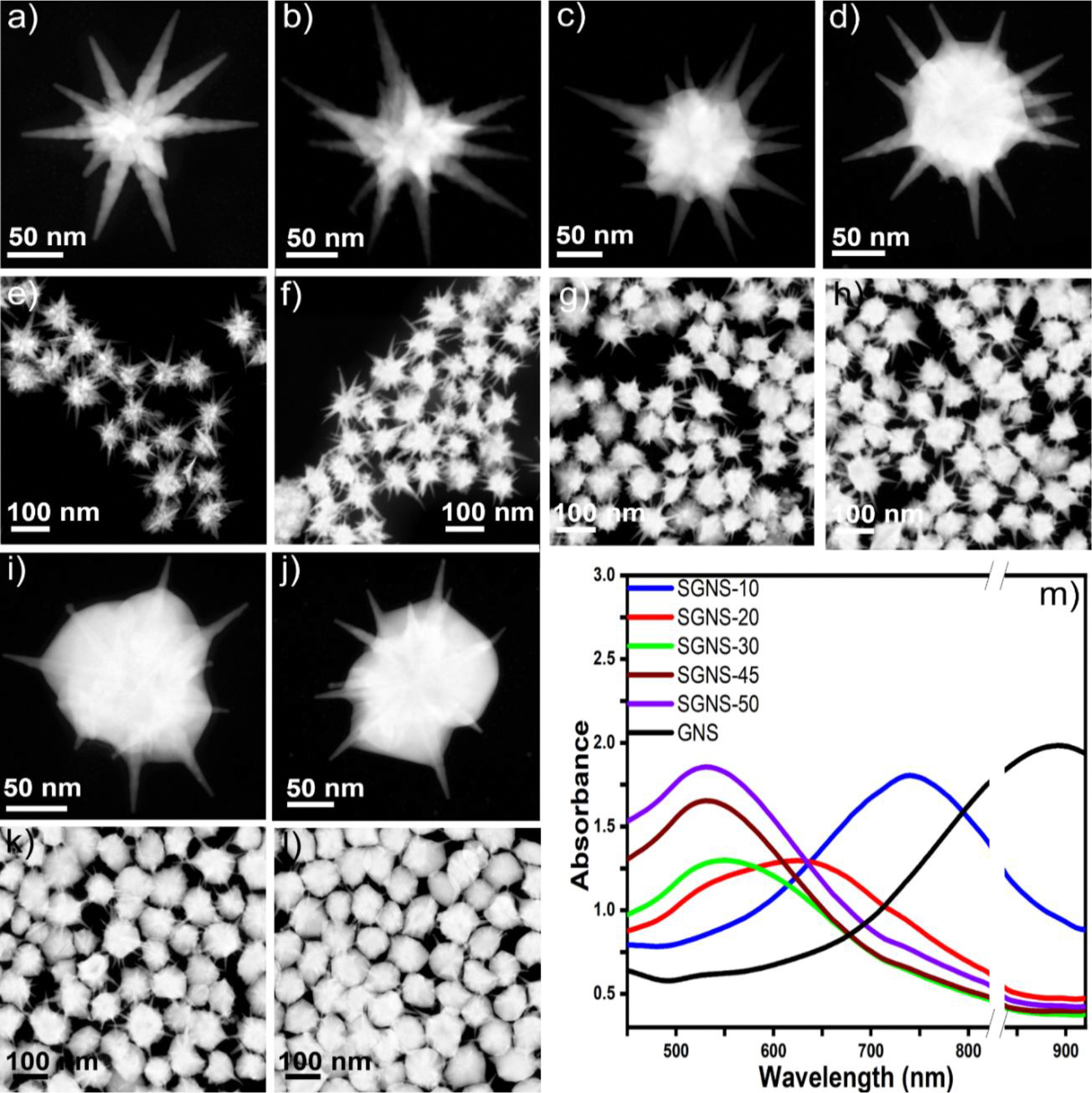
STEM images of GNSs (a), SGNS-10 (b), SGNS-20 (c), SGNS-30 (d), SGNS-45 (i), and SGNS-50 (j), indicating that high monodispersity of synthesis is achieved with retained sharply spiked morphology of GNS (e-h, k, and l). The UV-vis absorbance spectra of the GNSs and SGNSs indicate that the maximum plasmon resonance peak of the GNSs is blue shifted from 875 to 525 nm upon silver coating (m).

GNSs with a maximum number of sharp and long spikes were selected as a template for silver coating, which can result in the tunability and flexibility of the optical properties of SGNSs. In the first step, GNSs were synthesized using a surfactant-free seed-mediated synthesis procedure, which has advantages over other surfactant-based methods, as the probability of formation of nucleation centers to grow spikes is higher for the surfactant-free method than that for other methods[31]. To achieve highly anisotropic GNSs with sharp and long spikes, we used 20-nm seeds instead of traditional 12-nm seeds, which could lead to the growth of multiple sharp spikes[32]. Figure 1a shows the scanning transmission electron microscopy (STEM) image of the GNSs, indicating a highly spiked morphology, where the average spike length is 90 ± 5 nm. In the second step, the GNSs were coated with silver. Figures 1b–d, i, and j show the morphology of SGNSs (SGNS-10, SGNS-20, SGNS-30, SGNS-45, and SGNS-50). Figures 1e–h, 1k, and l show that the nanoparticle morphology is highly monodispersed. The LSPR band of the GNSs was blue shifted from 875 to 525 nm, with an increase in the thickness of silver on the GNSs (Figure 1m).

Figures S1 and S2a–d show the EDS images of the GNS and SGNS nanostructures, which indicate that the silver is deposited on the core of the GNSs. The average thicknesses of silver were 10, 20, 30, 45, and 50 nm for SGNS-10, SGNS-20, SGNS-30, SGNS-45, and SGNS-50, respectively. Interestingly, the silver layer thickness increased gradually with an increase in the AgNO_3_ concentration and reached a maximum of up to 50 nm (Figure S2e). The silver layer thickness did not increase with an increase in the AgNO_3_ concentration from >1.05 mM. Furthermore, we investigated the stability of the nanoparticles, which were stored under ambient conditions (2°C-8°C). Figure S2f shows the morphology of SGNS-45 after 30 days of synthesis, indicating that the GNS morphology was retained and there was no aggregation of the nanoparticles.

### Finite element modeling of SGNSs

**Figure 2.**
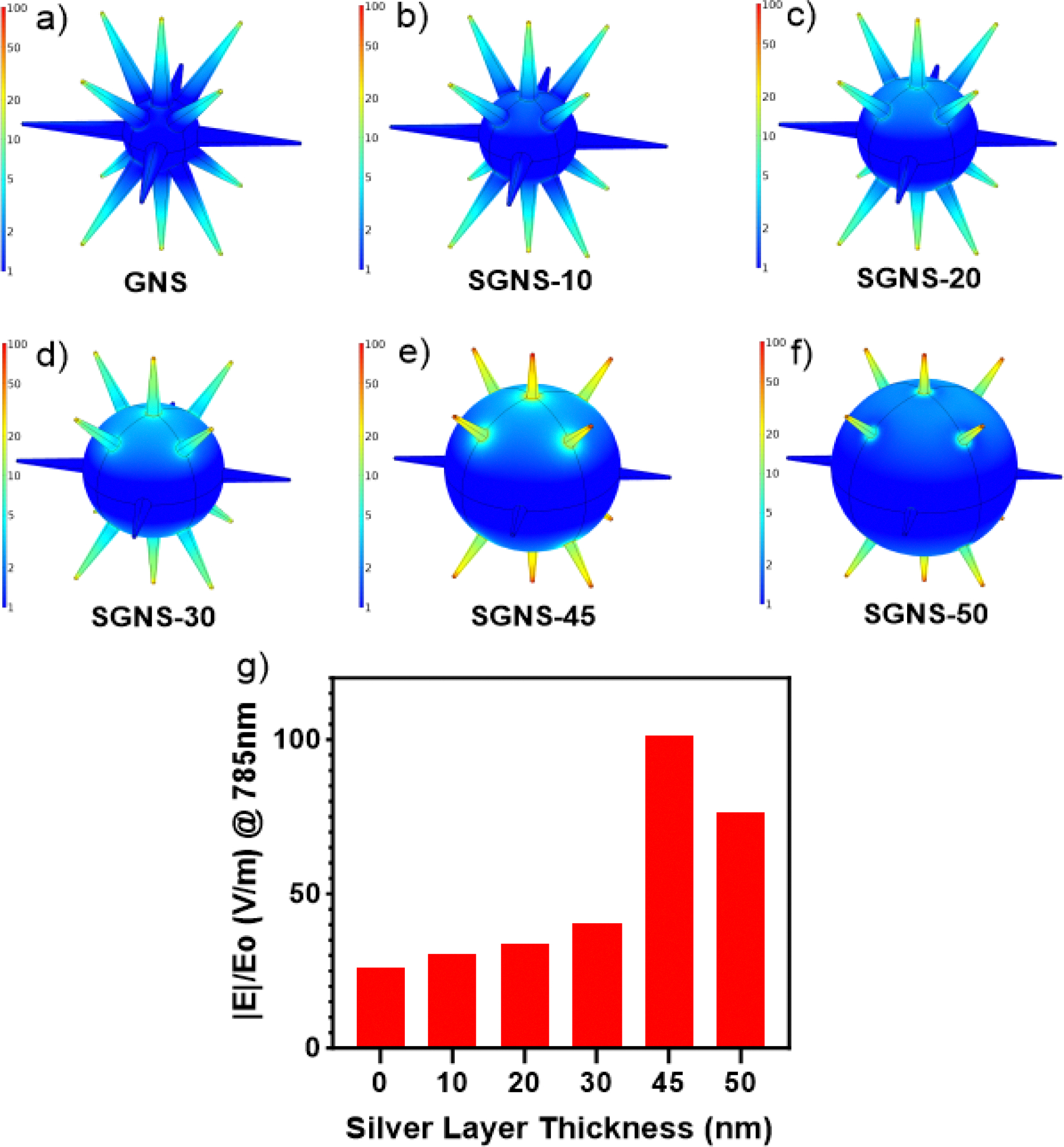
Normalized electric field |E|/|Eo| map at 785 nm for silver layer thicknesses of 0, 10, 20, 30, 43, and 65 nm (a-f). Maximum electric field enhancement |E|/|Eo| generated by each nanoparticle model at 785 nm, which indicates that the SGNS morphology with the 43-nm silver thickness possesses the highest electric field enhancement (g).

Furthermore, we investigated the electric field enhancement of the GNS morphology having different thicknesses of silver using the FEM simulation software, i.e., COMSOL Multiphysics 6.0, and the accompanying wave optics package to achieve the SGNS morphology, which would yield the greatest local electric field enhancement at 785 nm. To test the effect of the silver layer thickness on electric field enhancement, the underlying GNS model used herein was held constant, while the thickness of the silver layer varied to reflect the experimentally achieved morphologies shown in Figure 1. The base GNS model had a branch length of 90 nm, corresponding to the average experimentally observed dimensions. Figures 2a–f shows the nanoparticle model and the normalized electric field |E|/|Eo| generated at 785 nm for GNSs with silver layer thicknesses of 0, 10, 20, 30, 45, and 50 nm. In all the cases, maximum enhancement occurred at the branch tips. The maximum electric field enhancement observed for each simulated morphology is shown in Figure 2g. The magnitude of enhancement generated at the branch tips increased as the thickness of silver increased, where a maximum field enhancement of 101.38 V/m was observed with a silver thickness of 45 nm. Increasing the silver thickness further from 45 nm caused a subsequent decrease in branch tip enhancement.

### SERS performance of SGNSs

**Figure 3.**
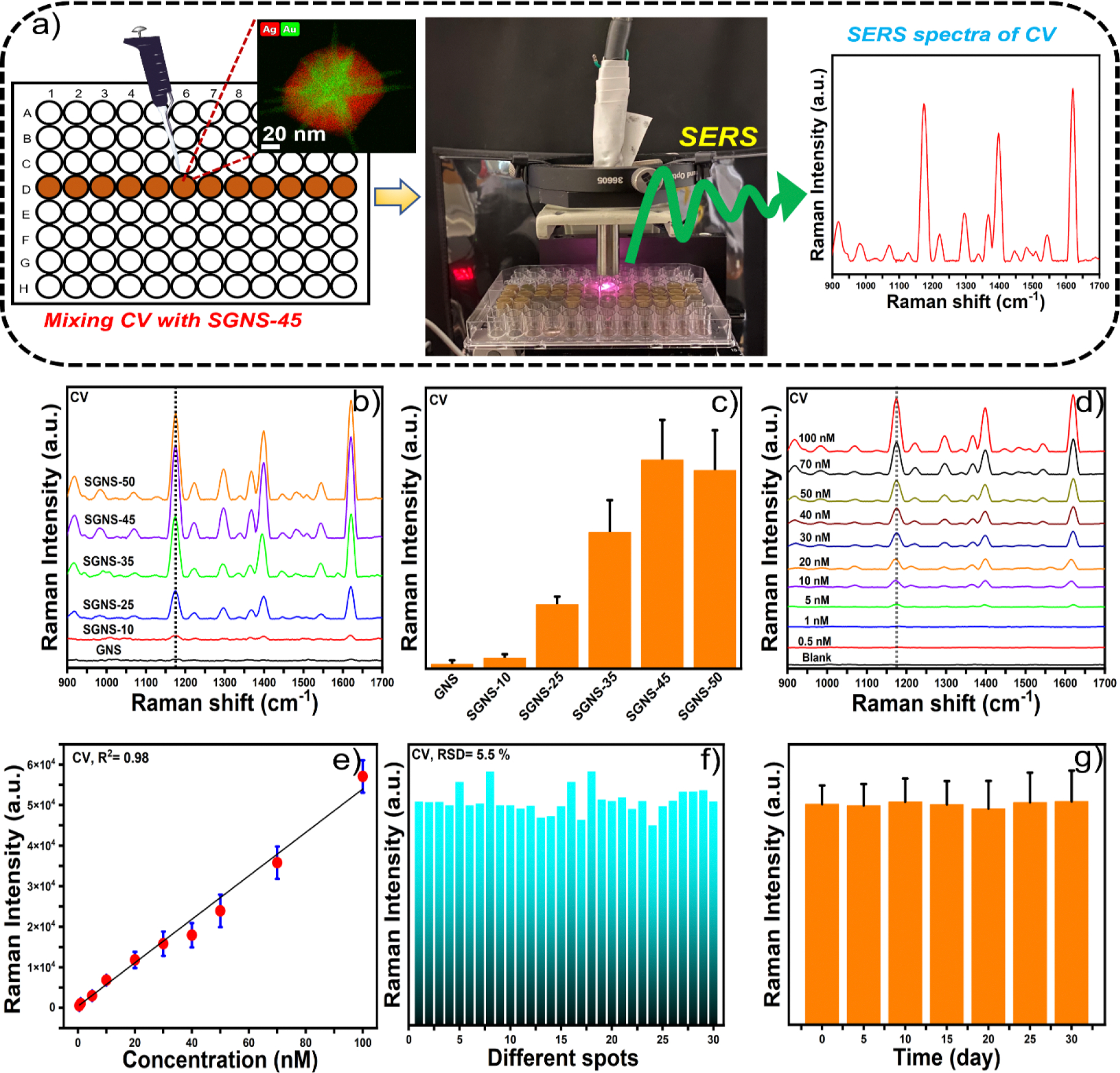
Microplate reader based on the high-throughput SERS platform assay (a). The SERS spectra of CV at 50 nM with different morphologies of SGNSs, which indicate that SGNS-45 exhibits the maximum SERS enhancement (b). The SERS intensity of CV at 1,174 cm^−1^ for different morphologies of SGNSs (c). The SERS spectra and the corresponding calibration plot of CV with SGNS-45 at different concentrations ranging from 100 to 0.5 nM (d and e). Reproducibility and stability of SGNS-45 (f and g).

Next, we investigated the SERS performance of the SGNS nanostructures to correlate with the simulation work. Herein, we considered microplate-based SERS measurement to achieve high-throughput SERS sensing of the analytes and a portable Raman system to achieve a rapid onsite SERS-sensing platform. Figure 3a shows the high-throughput SERS measurement. The application of the SERS substrate was first investigated using CV as a model analyte molecule. Figure 3b shows the SERS spectra of CV at 50 nM with GNSs and SGNSs. Figure 3c displays the Raman intensity of CV at 1,174 cm^−1^, which is attributed to the in-plane ring stretching of C-H vibrations [33]. The result shows that SGNS-45 exhibited the maximum SERS enhancement compared to other morphologies. SGNS-45 possessed the maximum number of hot spots and an optimal silver layer to achieve the maximum electric field, which almost matched the simulated results illustrated in Figure 2g.

The SERS measurements were further performed with the highest-performing nanoparticle system (SGNS-45) at different concentrations of CV, ranging from 100 to 0.5 nM (Figure 3d). Figure 3f shows the calibration plot of CV, which exhibits a linear relationship between the SERS intensity at 1,174 cm^−1^ and the CV concentration. The LOD of CV was calculated to be 0.1 nM with a good signal-to-noise ratio (S/N = 3.5). To achieve a reproducible microplate-based SERS platform, the SERS measurements of CV were performed in 30 different spots. The relative standard deviation (RSD) of the spot-to-spot SERS intensity at 1,174 cm^−1^ is as low as 5.5%, demonstrating that our microplate-based SERS platform possesses excellent signal reproducibility. Furthermore, the SERS stability of SGNS-45 was investigated (Figure 3g), which indicated that the peak intensity of CV did not change significantly over 30 days, implying the long-term stability of SGNS-45. Therefore, by synthesizing the SGNS morphologies and calculating the electric field distributions via finite element simulations, we were able to correlate the electric field intensity of the SGNS morphology with SERS signal enhancement, and the proposed micro-plate-based SERS platform exhibited good reproducibility and stability and is a potential candidate for practical real-life applications.

**Figure 4.**
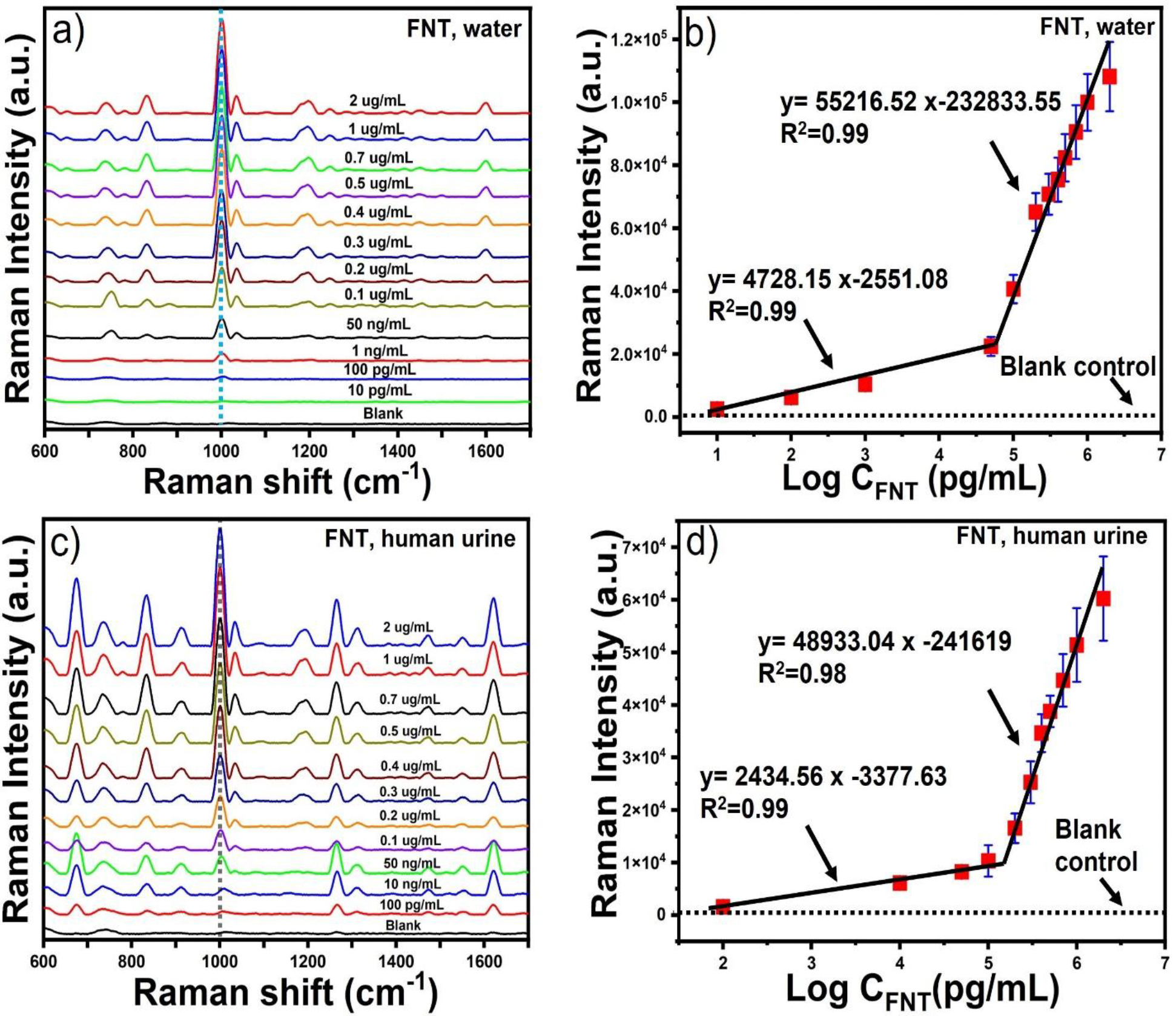
The SERS spectra of FNT in an aqueous solution at different concentrations, ranging from 2 μg/mL to 10 pg/mL (a). Calibration plot of FNT at 1,004 cm^−1^ against the logarithm value of the concentration of FNT (b). The SERS spectra of FNT spiked in human urine sample at different concentrations, ranging from 2 μg/mL to 100 pg/mL (c). Calibration plot of FNT at 1,004 cm^−1^ against the logarithm value of the concentration of FNT (d).

Having determined the optimal morphology for the SERS sensing of CV, we further investigated the quantitative SERS sensing of FNT using SGNS-45. Figure 4a depicts the SERS spectra of FNT in an aqueous solution for different concentrations ranging from 2 μg/mL to 100 pg/mL using SGNS-45, where the main characteristic peaks of FNT are located at 1,004 and 1,034 cm^−1^, which are attributed to the symmetric and antisymmetric phenyl ring breathing modes, respectively [14]. The Raman band at 1,004 cm^−1^ was selected as a calibration band to determine the calibration plot of FNT. There were two linear regions in the concentration ranges from 2 to 0.1 μg/mL and 50 ng/mL to 10 pg/mL that can be fitted with the straight lines y= 55216.52 log C_FNT_ (pg/mL) −232833.55 (R^2^ = 0.99) and y= 4728.15 log C_FNT_ (pg/mL) −2551.08 (R2 = 0.99), respectively, where y is the SERS intensity of FNT (Figure 4b). The presence of two linear regions of the calibration plots is consistent with the previously reported studies[34, 35]. We determined the limit of detection (LOD) according to the IUPAC definition: LOD = 3σ/S [34], where σ and S represent the standard deviation of blank measurements and the slope of the linear equation in the low concentration region of 50 ng/mL to 10 pg/mL, respectively. Low levels of FNT (LOD) were detected as low as 5.54 pg/mL.

There is a growing concern about illegally misusing the mixture of opioids with FNT to make it even stronger in euphoric and hallucinogenic effects. Therefore, it is important to detect FNT and FNT mixed with other opioids. We have studied the SERS detection of FNT laced in opioid codeine using the proposed microplate-based high-throughput SERS platform, where a codeine stock solution (10 μg/mL) was mixed with the FNT solution at different mass concentrations ranging from 1% to 10%. Figure S3a shows the SERS spectra of FNT laced in codeine, where the characteristic peak appeared at 620 cm^−1^ attributed to the C-C bending mode of codeine [36]. Figure S3b shows the corresponding calibration curve plotted against the peak ratio of FNT at 1,004 cm^−1^ with the mass concentration ranging from 1% to 10% to codeine. In addition, we have applied this high-throughput SERS platform for the detection of a ternary mixture of opioids: codeine, morphine, and FNT. Figure S3c shows the SERS spectra of individual drugs (FNT at 500 ng/ mL, morphine at 10 μg/mL, and codeine at 10 μg/mL) and the mixture of them at the top. Altogether, this study demonstrates that the proposed microplate-based SERS platform is capable of trace detection of FNT in a binary or ternary mixture of opioids.

### Detection of FNT in human urine

SERS is a very chemical-specific technique that exhibits multiple sharp Raman peaks associated with the molecular vibrations of the target analytes. For this reason, the SERS-Raman spectra are often referred to as “spectral fingerprints” of analyte molecules. To validate our detection method for real-life applications, we have studied the SERS measurements of FNT with SGNS-45 nanoparticles, where FNT was spiked in human urine samples. We first investigated the specificity and selectivity of the SERS peaks of FNT with blank having pooled human urine samples and a healthy volunteer’s urine sample from Duke University. As depicted in Figure S4, the characteristic peaks of FNT at 1,004 and 1,034 cm^−1^ can be clearly distinguished from the characteristic peaks of human urine at 735, 1013, and 1265 cm^−1^ which are assigned to cholesterol, urea, and cytosine, respectively [37]. The results indicated the high selectivity of our SERS method for FNT detection. Figure 4c shows the SERS spectra of FNT with a concentration range of 2 μg/mL to 100 pg/mL. There were two linear regions in the concentration ranges from 2 to 0.2 μg/mL and 0.1 μg/mL to 100 pg/mL that can be fitted with the straight lines y= 48933.04 log C_FNT_ (pg/mL) −241619 (R^2^ = 0.98) and y= 2434.56 log C_FNT_ (pg/mL) −3377.63 (R2 = 0.99), respectively, where y is the SERS intensity of FNT (Figure 4d). We determined the limit of detection (LOD) according to the IUPAC definition: LOD = 3σ/S, where σ and S represent the standard deviation of blank measurements and the slope of the linear equation in the low concentration region of 0.1 μg/mL to 100 pg/mL, respectively. The LOD was calculated as low as 10.02 pg/mL. We further determined the limit of detection (LOQ) according to the IUPAC definition: LOQ = 10σ/S, where σ and S represent the standard deviation of blank measurements and the slope of the linear equation in the low concentration region of 0.1 μg/mL to 100 pg/mL, respectively. The LOQ was calculated as low as 28.5 pg/mL. We investigated the FNT concentration range that is clinically relevant. The clinical FNT overdose concentration in human urine has been reported to range from 5 to 95 ng/mL[38]. This indicates that our SERS detection method can be utilized for rapid sensitive FNT overdose detection. To verify the reliability and practicability of this method, the recovery percentage of FNT was investigated by spiking FNT into human urine of three different concentrations (Table S1). We calculated the recovery (C2/C1×100%) of FNT, where C2 is the spiked FNT concentration and C1 is the observed FNT concentration[34]. According to Table S1, the recovery percentage of all samples are from 92.5 % to 102 % with relative standard deviations (RSD) of less than 10 %.

We compared the measurement results with the results of other SERS-sensing approaches (Table S2), indicating that the proposed method is approximately 100 times more sensitive compared to the reported values related to the detection of FNT in biological samples without any sample preprocessing. Moreover, the proposed SERS platform helped identify FNT (500 ng/mL) laced in codeine (10 μg/mL) spiked in human urine, indicating the possibility of the practical trace detection of FNT in binary mixtures of opioids (Figure S5). In summary, we demonstrated the most effective morphology of the silver-coated SGNSs (SGNS-45) via experimentation and theoretical simulation to achieve maximum electric field enhancement, and then, we used it for quantitative ultrahigh FNT detection in human urine without any sample preparation, in contrast to conventional multistep analyte extraction and detection approaches.

## Conclusion

We developed an SGNS-based SERS-sensing platform for the rapid ultrasensitive trace detection of FNT in human urine using a portable Raman instrument. We synthesized sharply spiked GNSs with an optimum layer of silver to achieve maximum SERS enhancement. We successfully validated the SERS enhancement through experiments and theoretical simulations of SGNSs. The hybrid structure, combining sharp protruding tips and the high plasmonic effect of silver coating, resulted in high SERS enhancing performance. Moreover, we employed the proposed SERS-sensing platform for the trace detection of FNT in an aqueous solution and human urine. In contrast to conventional analytical methods such as HPLC, GC-MS, and ELISA, our SERS assay is straightforward, rapid, and direct delivery of analytes to the SGNS-45 solution and subsequent SERS measurements in 0.5 seconds. Overall, this study highlights the key factors effective for the generation of enhanced electric fields in silver-coated SGNSs and their potential for the sensitive detection of FNT in human urine samples, thereby opening the possibility for rapid onsite monitoring of illegal street drugs.

## Supplementary Material

Supporting Information
