# Supplementary material for: Rapid SERS Assay for Determination of the Opioid Fentanyl Using Silver-Coated Sharply-Branched Gold Nanostars
Source: Mikrochim Acta. Author manuscript; Available in PMC 2026 Jan 6. (PMC12768890; doi:10.1007/s00604-023-06172-5)
Supplement: Supporting Information [file NIHMS2126900-supplement-Supporting_Information.docx]

**SUPPLEMENTARY INFORMATION (SI)**

**Rapid SERS Assay for Determination of the Opioid Fentanyl Using Silver-Coated Sharply-Branched Gold Nanostars**

*Supriya Atta, ^a, b^ Aidan J. Canning, ^a, b^ Tuan Vo-Dinh ^a,^ ^b, c‡^*

^a^ Fitzpatrick Institute for Photonics, ^b^ Department of Biomedical Engineering, ^c^ Department of Chemistry, Duke University, Durham, NC 27708, USA.

**Synthesis of SGNS**

We have first synthesized GNS following a modified reported method[1]. We have used 20 nm seeds for the GNS synthesis, and it was synthesized by following a reported procedure[2]. The synthesis of gold nanostars is described below. 50 mL of 1 mM HAuCl_4_ solution was first prepared. After that, 200 μL of 1 M HCl and 500 µL of 20 nm was added to a solution. Then, 2 mL of 3 mM AgNO_3_ and 1 mL of 100 mM ascorbic acid were added simultaneously to the solution. The solution was stirred for 2 minutes and immediately used for silver coating of gold nanostars.

SGNS was synthesized by following our previously reported method with small modification, where a certain amount of milli-Q water was added first to the above mentioned GNS solution, and then a certain amount of AgNO_3_, and ascorbic acid were added simultaneously to the GNS solution[3]. Briefly, for SGNS-10, 48 mL milli-Q water was added first to the GNS solution, and then 1 mL 15 mM AgNO_3_ and 1 mL 100 mM ascorbic acid were added simultaneously to the GNS solution. For SGNS-20, 44 mL milli-Q water was added first to the GNS solution, and then 3 mL 15 mM AgNO_3_ and 3 mL 100 mM ascorbic acid were added simultaneously to the GNS solution. For SGNS-30, 40 mL milli-Q water was added first to the GNS solution, and then 5 mL 15 mM AgNO_3_ and 5 mL 100 mM ascorbic acid were added simultaneously to the GNS solution. For SGNS-45, 36 mL milli-Q water was added first to the GNS solution, and then 7 mL 15 mM AgNO_3_ and 7 mL 100 mM ascorbic acid were added simultaneously to the GNS solution. For SGNS-50, 32 mL milli-Q water was added first to the GNS solution, and then 9 mL 15 mM AgNO_3_ and 9 mL 100 mM ascorbic acid were added simultaneously to the GNS solution. The solution was stirred overnight to grow the silver layer on the GNS and then it stored at 2-8° C.

**Raman Measurements**

The Raman measurements were studied by using a lab-made portable Raman instrument with a 785 nm laser source. The laser power and the exposure time was used 200 mW and 0.5 sec, respectively. We used 96 well plate for SERS measurement study, which was covered with an aluminum foil to avoid the signal effect of the plastic. The FNT-spiked human urine sample has been prepared as follows. In the first step, we prepared a certain concentration of FNT in methanol. We then mixed the FNT solution in human urine samples at a 1:1 ratio without purification. For a typical SERS measurement, 3μL of FNT-human urine mixture solution was mixed with 297 μL of as synthesized SGNS-45 nanoparticle solution. We have recorded the final concentration of the FNT to plot the calibration curve.


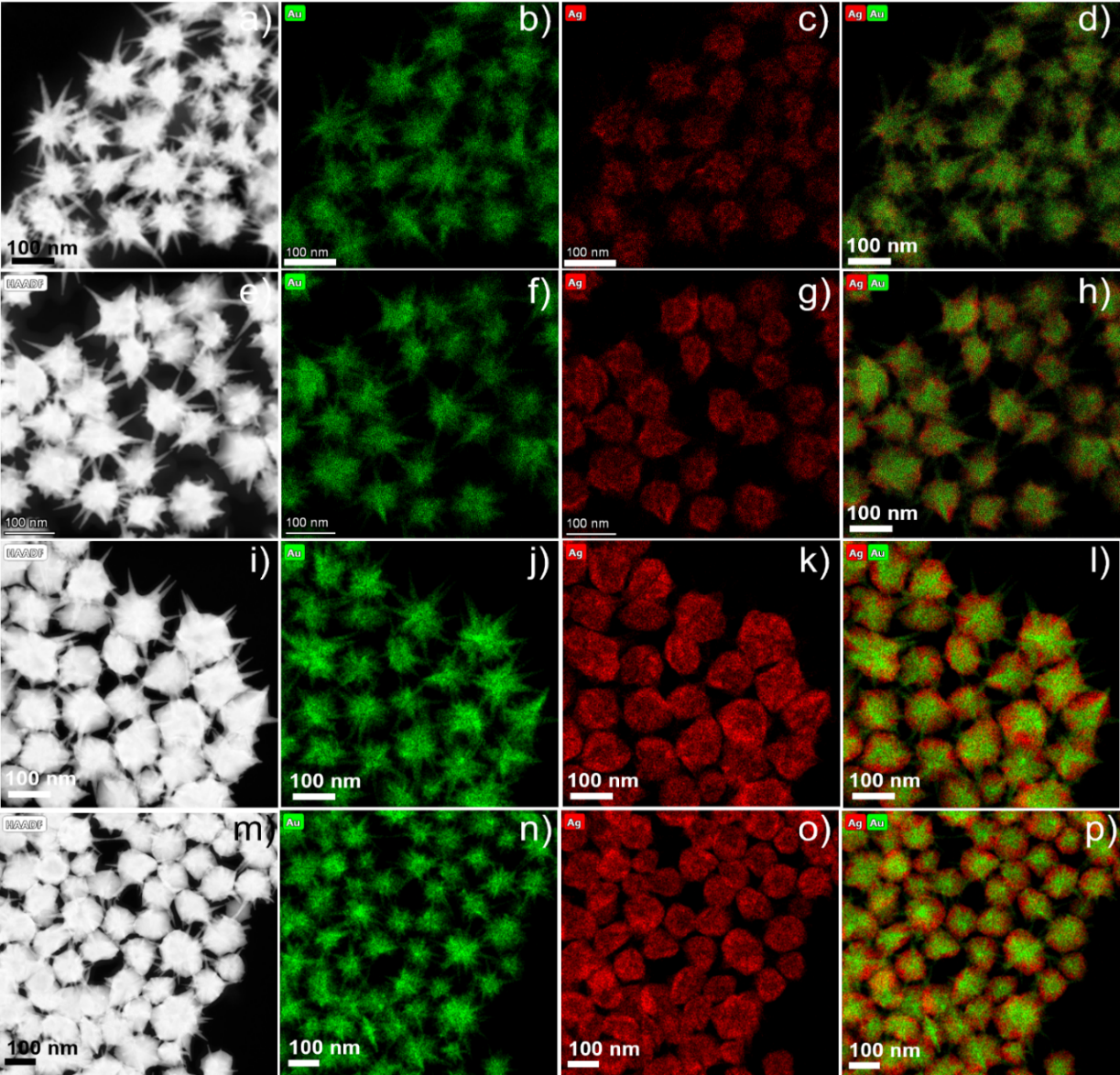


**Figure S1.** EDS elemental mapping of SGNS-10 (a-d), SGNS-20 (e-h), SGNS-30 (i-l), and SGNS-50 (m-p) showing silver layer is deposited on the core of GNS.


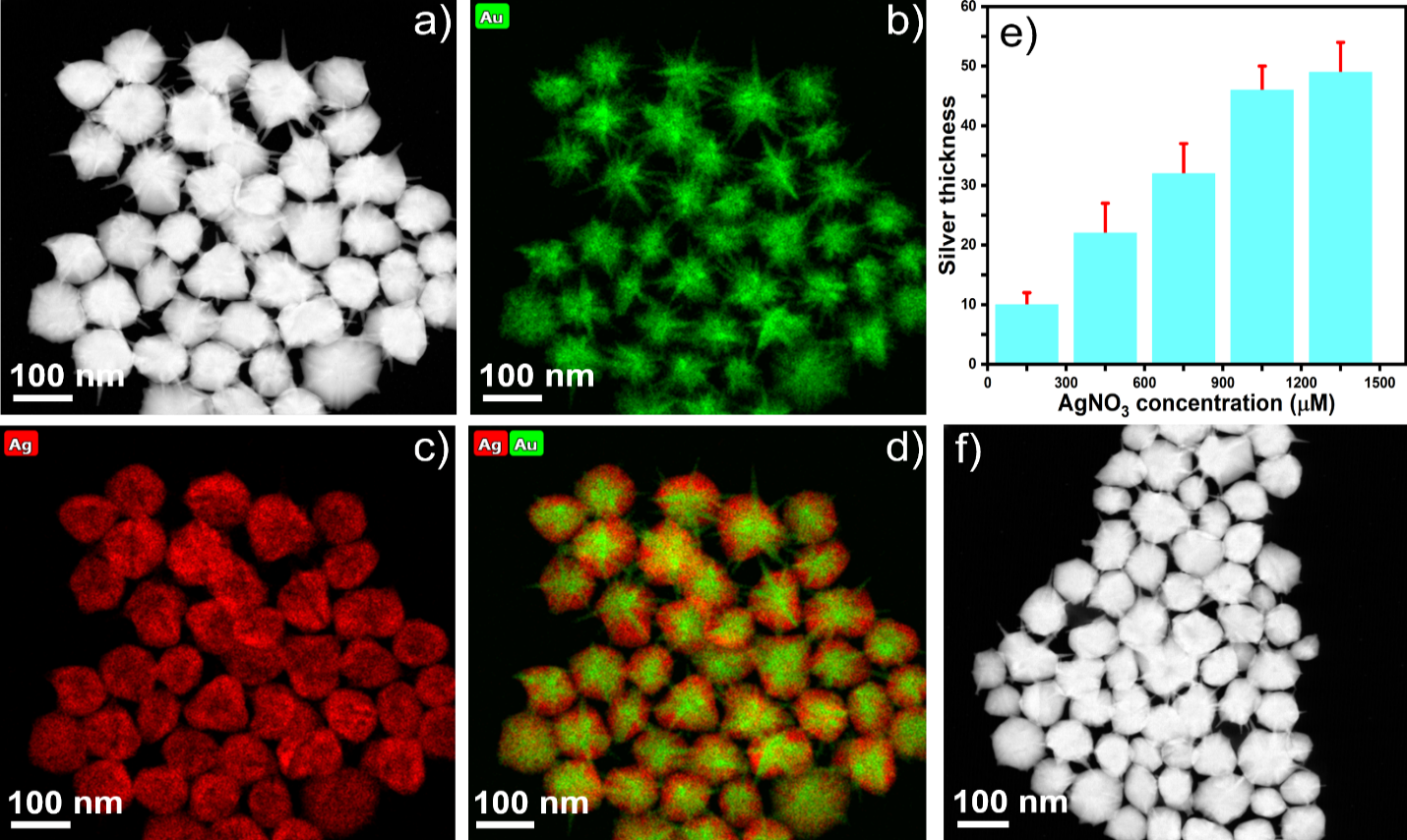
**Figure S2.** STEM-EDS images of SGNS-45 (a-d) indicate that the silver is preferentially deposited on the core of GNSs. (e) Increment in the silver thickness with an increase in the AgNO_3_ concentration. STEM image of SGNS-45 after 30 days of synthesis, indicating that the GNS morphology is retained (f).


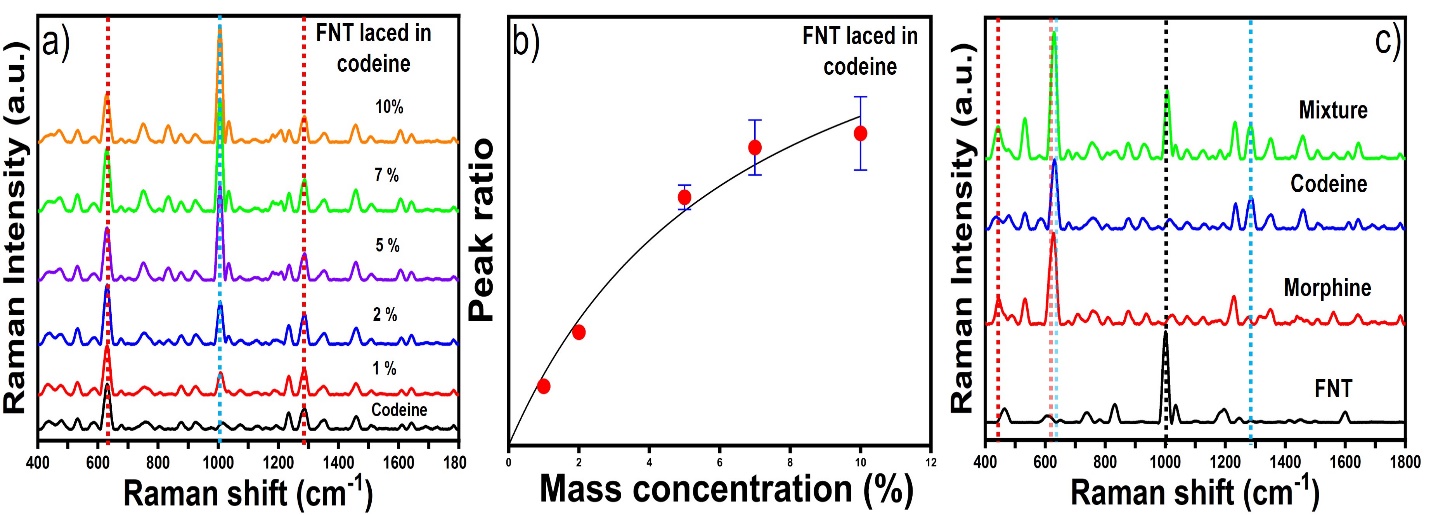


**Figure S3.** The SERS spectra of FNT laced in codeine at different mass concentrations, ranging from 1% to 10%, and the corresponding calibration curve plotted against the peak ratio of FNT at 1,004 cm^−1^ and codeine at 628 cm^−1^ with the mass concentration ranging from 1% to 10% with respect to codeine (a-b). SERS spectra of individual drugs (FNT at 500 ng/ mL, morphine at 10 µg/mL, and codeine at 10 µg/mL) and a mixture of them at the top (c).


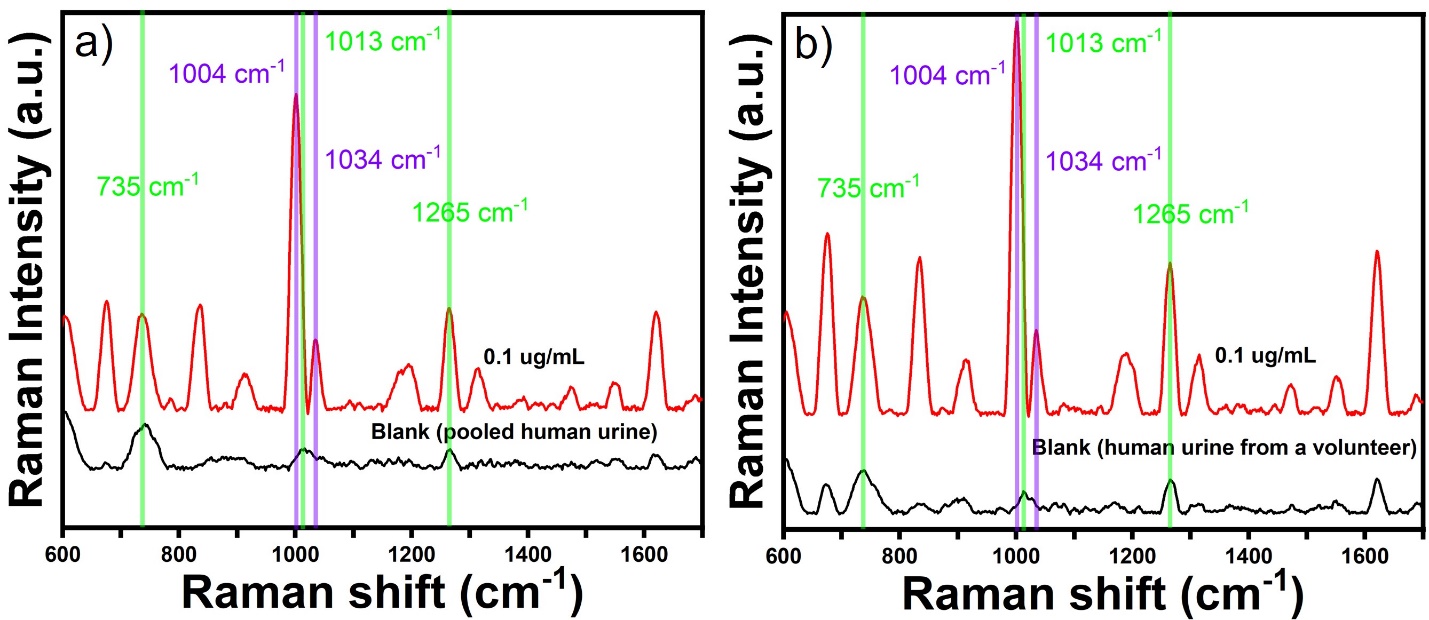
 **Figure S4.** The SERS spectra of pooled human urine (black line), and 100 pg/mL FNT spiked in pooled human urine (red line) (a). The SERS spectra of a healthy volunteer’s urine at Duke University (black line), and 0.1 µg/mL FNT spiked in human urine (red line) (b).


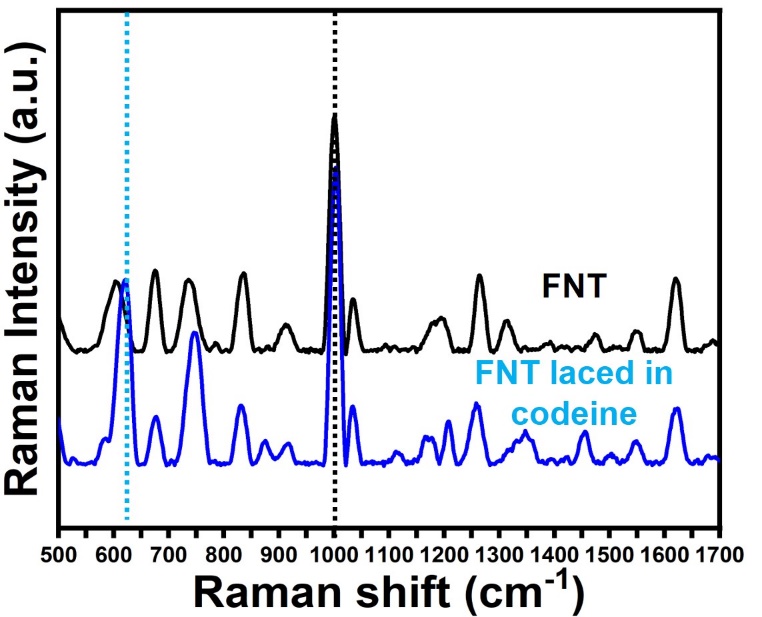


**Figure S5.** The SERS spectra of FNT and FNT (500 ng/mL) laced in codeine (10 µg/mL) spiked in human urine (e).

**Table S1.** Recovery percentage of SERS measurement of FNT in human urine samples. *

| Spiked Concentration (g/mL) | Observed Concentration  (g/mL) | Recovery (%) | RSD (%) |
| --- | --- | --- | --- |
| 1 × 10^-6^ | 0.925 × 10^-6^ | 92.5 | 8.8 |
| 0.5×10^-6^ | 0.51×10^-6^ | 102.0 | 7.5 |
| 10×10^-9^ | 9.901×10^-9^ | 99.0 | 9.2 |

* The measurements were repeated three times each.

**Table S2.** Literature survey for FNT detection by SERS.

| Limit of detection (LOD) ng/mL | Linear ranges | Recovery value (%) | Type of Nanoparticles | Type of SERS substrate | References |
| --- | --- | --- | --- | --- | --- |
| 5 ng/mL (artificial urine) | 0 to 100 ng/mL | - | Silver nanoparticles | Solution-based (salt-induced aggregation of nanoparticles) | [4] |
| 1.86 ng/mL (water)  40.63 ng/mL (serum) | 0 to 2 µg/mL | - | Gold-Trisoctahedra | Capillary-based | [5] |
| 100 ng/mL | Linearity was observed at < 6% fentanyl | - | Silver nanoparticle | Paper-based substrate | [6] |
| 33.64 mg/mL | - | - | Silver nanoparticles | Microfluidic device (salt-induced aggregation of nanoparticles) | [7] |
| 6 ng/mL | - | - | Gold nanoparticle | SERS flow strip | [8] |
| 0.47 ng/mL (serum)  0.73 ng/mL (urine) | Two linear range- 0 to 300.0 ng/mL, and 0.8 to 50.0 µg/mL | 85.0-93.7% | GNSs | Hydrophobic plasmonic paper | [9] |
| 0.46 ng/mL (water)  2.31 ng/mL (urine) | 5-200 ng/mL | 90.21-109.87 % | MOF-gold core-satellite nanostructure | Solution-based | [10] |
| 1 ng/mL (water)  50 ng/mL (urine) | 1 to 100 ng/mL | - | Gold nanoparticles | liquid/liquid interfacial plasmonic arrays | [11] |
| 0.00554 ng/mL (water)  0.01002 ng/mL (urine) | Two linear range-2 to 0.1 µg/mL and 50 ng/mL to 100 pg/mL | 92.5-102 % | SGNS-45 | High-throughput microplate-based platform-**This work** | **This work** |

**References**

[1] S. Atta, T. Watcharawittayakul, T. Vo-Dinh, Ultra-high SERS detection of consumable coloring agents using plasmonic gold nanostars with high aspect-ratio spikes, Analyst 147(14) (2022) 3340-3349.

[2] K. Sugawa, T. Akiyama, Y. Tanoue, T. Harumoto, S. Yanagida, A. Yasumori, S. Tomita, J. Otsuki, Particle size dependence of the surface-enhanced Raman scattering properties of densely arranged two-dimensional assemblies of Au(core)–Ag(shell) nanospheres, Physical Chemistry Chemical Physics 17(33) (2015) 21182-21189.

[3] S. Atta, T. Vo-Dinh, Bimetallic Gold Nanostars Having High Aspect Ratio Spikes for Sensitive Surface-Enhanced Raman Scattering Sensing, ACS Applied Nano Materials 5(9) (2022) 12562-12570.

[4] H. Wang, Z. Xue, Y. Wu, J. Gilmore, L. Wang, L. Fabris, Rapid SERS Quantification of Trace Fentanyl Laced in Recreational Drugs with a Portable Raman Module, Analytical Chemistry 93(27) (2021) 9373-9382.

[5] M. Zhang, J. Pan, X. Xu, G. Fu, L. Zhang, P. Sun, X. Yan, F. Liu, C. Wang, X. Liu, G. Lu, Gold-Trisoctahedra-Coated Capillary-Based SERS Platform for Microsampling and Sensitive Detection of Trace Fentanyl, Analytical Chemistry 94(11) (2022) 4850-4858.

[6] A. Haddad, M.A. Comanescu, O. Green, T.A. Kubic, J.R. Lombardi, Detection and Quantitation of Trace Fentanyl in Heroin by Surface-Enhanced Raman Spectroscopy, Analytical Chemistry 90(21) (2018) 12678-12685.

[7] R. Mirsafavi, M. Moskovits, C. Meinhart, Detection and classification of fentanyl and its precursors by surface-enhanced Raman spectroscopy, Analyst 145(9) (2020) 3440-3446.

[8] C. Shende, C. Brouillette, S. Farquharson, Detection of codeine and fentanyl in saliva, blood plasma and whole blood in 5-minutes using a SERS flow-separation strip, Analyst 144(18) (2019) 5449-5454.

[9] X. Su, X. Liu, Y. Xie, M. Chen, H. Zhong, M. Li, Quantitative Label-Free SERS Detection of Trace Fentanyl in Biofluids with a Freestanding Hydrophobic Plasmonic Paper Biosensor, Analytical Chemistry 95(7) (2023) 3821-3829.

[10] X. Li, L. Sun, B. Xu, L. Dai, Y. Xiao, Y. Ding, Q. Liu, M. Meng, R. Xi, L. Guo, Y. Yin, MOF-gold core-satellite nanostructure based SERS platform for fentanyl detection in multiple complex samples, Sensors and Actuators B: Chemical 385 (2023) 133710.

[11] Z. Ding, C. Wang, X. Song, N. Li, X. Zheng, C. Wang, M. Su, H. Liu, Strong π-Metal Interaction Enables Liquid Interfacial Nanoarray–Molecule Co-assembly for Raman Sensing of Ultratrace Fentanyl Doped in Heroin, Ketamine, Morphine, and Real Urine, ACS Applied Materials & Interfaces 15(9) (2023) 12570-12579.
